# Leptospirosis: Updating the Global Picture of an Emerging Neglected Disease

**DOI:** 10.1371/journal.pntd.0004039

**Published:** 2015-09-24

**Authors:** Mathieu Picardeau

**Affiliations:** Unité de Biologie des Spirochètes, Institut Pasteur, Paris, France; University of Tennessee, UNITED STATES

Leptospirosis is a zoonotic bacterial disease found predominantly in impoverished populations inhabiting developing countries with tropical climates. Global leptospirosis burden is significant; however, inadequate diagnosis has affected the awareness of the disease among the medical community. This issue of *PLOS Neglected Tropical Diseases* reports two studies commissioned by the Leptospirosis Burden Epidemiology Reference Group (LERG) of the World Health Organization (WHO) on evaluating the burden of leptospirosis and determinants of transmission, based on systematic reviews of currently available literature.

The first and long-awaited paper, by Costa et al. [1], updates previously published estimates from 1999, which relied on national surveys of surveillance data from different parts of the world [2]. In the present study, leptospirosis is estimated to cause more than 1 million severe cases and approximately 60,000 deaths per year. The limitations of surveillance systems in low-income tropical countries likely contribute to an underestimation of its burden. This is the case for numerous countries in Africa, for example, where there is increasing evidence that leptospirosis accounts for a significant proportion of nonmalarial fever cases. The burden of leptospirosis is therefore comparable or even higher than some other important neglected tropical diseases, including visceral leishmaniasis, severe dengue, echinococcosis, and cysticercosis.

In the second paper, by Mwachui et al. [3], an overview of risk factors for leptospirosis is presented. Leptospirosis is a complex disease with multiple modes of transmission, broad host range, a multitude of infecting serovars, nonspecific clinical manifestation, and difficult diagnosis. The authors found a high heterogeneity of risk factors among the selected studies, suggesting that epidemiological patterns are highly specific to the geo-climatic context. Water-associated exposures (recreational water activities in developed countries and floods and heavy seasonal rainfall in tropical countries) are the main risk factors. Other risk factors include, as expected, agricultural practices, contact with animals, and poor sanitation. The burden of leptospirosis appears to be mainly determined by the interaction of poverty, geography, and climate.

Both papers emphasize the scarcity of data on the surveillance and epidemiology of leptospirosis, as well as a lack of consensus on definitions of cases and risk factors, study design, and adequate methods of data analysis. There is a need for new studies with stringent epidemiological and diagnostic criteria, especially in countries that are not investing in surveillance programs. Further progress in our understanding of the epidemiology should contribute to the development of effective intervention strategies to prevent and control outbreaks. This is the challenge the Global Leptospirosis Environmental Action Network (GLEAN) from the WHO and the Health and Climate Foundation has been tasked with, through an interdisciplinary One Health approach (http://www.glean-lepto.org). Also, Leptospirosis is an emerging disease because of the growing number of inhabitants residing in urban slums and increased frequency of extreme climatic events. Although not included among the 17 NTDs prioritized by WHO (http://www.who.int/neglected_diseases/diseases/en/), these two papers should contribute to raising awareness on leptospirosis as an important and emerging neglected tropical disease.
